# Perceived Barriers and Facilitators in Using Patient-Reported Outcome Systems for Cancer Care: Systematic Mapping Study

**DOI:** 10.2196/40875

**Published:** 2023-06-28

**Authors:** Anna-Mari Laitio, Guido Giunti, Raija Halonen

**Affiliations:** 1 Faculty of Information Technology and Electrical Engineering University of Oulu Oulu Finland; 2 Faculty of Medicine University of Oulu Oulu Finland; 3 School of Medicine Trinity College Dublin Dublin Ireland

**Keywords:** patient-reported outcome system, barriers, facilitators, cancer, health care professionals

## Abstract

**Background:**

Cancer is a major global health problem. Patient-reported outcome (PRO) systems have been developed to support the treatment of patients with cancer. Although clear evidence of the benefits of the routine use of electronic patient-reported outcomes (ePROs) exists, engaging physicians in using these systems has been challenging.

**Objective:**

This study aims to identify and analyze what is currently known about health care professionals’ (HCPs) perceived barriers and facilitators that exist and influence the use of ePRO systems for cancer care.

**Methods:**

We carried out a systematic mapping study by conducting searches of 3 databases (Association for Computing Machinery, PubMed, and Scopus). Eligible papers were published between 2010 and 2021, and they described HCPs’ perspectives on using ePROs. The data on the included papers were extracted, a thematic meta-synthesis was performed, and 7 themes were summarized into 3 categories.

**Results:**

A total of 17 papers were included in the study. The HCPs’ perceived barriers and facilitators of using ePROs can be summarized into 7 themes: clinical workflow, organization and infrastructure, value to patients, value to HCPs, digital health literacy, usability, and data visualization and perceived features. These themes can be further summarized into 3 categories: work environment, value to users, and suggested features. According to the study, ePROs should be interoperable with hospital electronic health records and adapted to the hospital workflow. HCPs should get appropriate support for their use. Additional features are needed for ePROs, and special attention should be paid to data visualization. Patients should have the option to use web-based ePROs at home and complete it at the time most valuable to the treatment. Patients’ ePRO notes need attention during clinical visits, but ePRO use should not limit patient-clinician face-to-face communication.

**Conclusions:**

The study revealed that several aspects need improvement in ePROs and their operating environments. By improving these aspects, HCPs’ experience with ePROs will enhance, and thus, there will be more facilitating factors for HCPs to use ePROs than those available today. More national and international knowledge about using ePROs is still needed to cover the need for information to develop them and their operating environments to meet the needs of HCPs.

## Introduction

Cancer is one of the most important health problems, affecting nearly 25 million people globally each year through new cancer incidences [1]. Routine patient-reported outcome (PRO) follow-up of patients with cancer improves long-term treatment outcomes [2,3]. Integrating PRO measures into routine clinical practice has improved symptom monitoring and the detection of treatment complications in patients with cancer [4]. Using PROs has resulted in fewer hospitalizations and emergency room visits, better health-related quality of life, and higher quality-adjusted survival [2]. Furthermore, PROs can improve communication between patients and health care professionals (HCPs) [4-6].

Patient-reported outcome measures (PROMs) are measurement tools used to report PROs. Nondigital and digital PROs (electronic patient-reported outcomes [ePROs]) use PROMs to collect PRO data [7]. ePRO is a software that allows patients to independently answer questions and report on their health using electronic devices, and HCPs can follow their patients’ well-being and assessment of symptoms. ePRO provides decision support for HCPs by helping with symptom monitoring and improving patient-clinician communication [8]. Although most physicians agree on the importance of collecting self-reported data of patients with cancer, engaging physicians in using PROs is a key challenge [9]. Furthermore, nurses and physicians have various preferences regarding PROMs in clinical practice [10]. Increasing the awareness of PRO solution providers regarding the barriers and facilitators to using PROs in clinical practice can help inform the design of these tools to support the enhancement of the quality of patient care [11]. Over the previous decade, research has focused on expanding our understanding of the benefits for patients of using PROs in cancer care [12].

The acceptability of PROM is often linked to its perceived benefits [13,14]. HCPs often support the use of PROMs that bring benefits to patients and improve health care [14]. However, formal integration of these tools into the hospital electronic health record (EHR) is infrequent [15], despite evidence that it improves the feasibility of their use [13]. It is important that the PROM is easy to navigate and that HCPs have easy access to computers and sufficient skills and knowledge to use the PROM. The relevance of workflow has also been highlighted as a significant aspect of the feasibility of PROM use [13]. Previous works in the literature also show other barriers and facilitators: patients’ limited eHealth literacy [16], lack of friendly interface elements for displaying longitudinal patient-reported symptoms, and integrations with EHRs [9].

At the time of this study, only a few studies have explored the barriers and facilitators of HCPs’ experiences when using ePRO systems to support the treatment of patients with cancer. The purpose of this study was to identify and analyze the current landscape on this topic.

## Methods

### Study Design

The study was carried out as a systematic mapping study to structure, understand, and organize existing research work on HCPs’ experience with ePRO systems [17,18]. Systematic reviews provide a synthesis of valuable studies in a particular field of research that is not possible for a practitioner to read on their own [19], while the systematic mapping study aims to structure the research area [18]. A mapping study is a practical method for a researcher who needs to understand and organize the existing research work in an individual domain [17].

### Data Exclusion

Keywords for this study included patient-reported outcomes, barriers, and cancer. We also used the MeSH terms neoplasms, patient-reported outcome measures, telemedicine, assessment, and patient outcomes as search terms. These keywords were combined with Boolean, and search results were narrowed by the publication date of the year 2010 onward to identify appropriate studies, as shown in Multimedia Appendix 1.

### Selection Criteria

Papers were included if (1) they were written in English, (2) the studies included the use of ePROs, (3) they were published between or during 2010 and 2021, (4) the target population included patients with cancer, and (5) they mentioned HCPs’ perspectives (barriers and facilitators) of using PROMs.

Papers were excluded if they were (1) focused on PROM use in clinical trials, (2) review studies, and (3) focused on the implementation of PROM for clinical practice.

For the purposes of this study, we considered the PROMs used in the past 3 months after the implementation period.

### Data Screening

**Figure 1:**
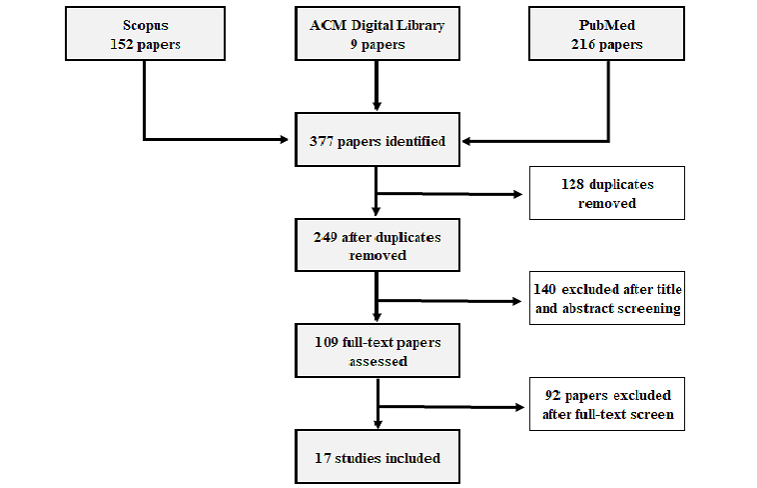
The number of included and excluded papers during the study selection process. ACM: Association for Computing Machinery.

Figure 1 illustrates the study selection process and shows the number of included and excluded papers. The papers were imported from 3 academic research databases to Covidence (Covidence.org). Covidence, a systematic review management tool, was used to remove duplicates and manage all the references included in the title and abstract screening, full-text review, and extraction. The searches from the databases were done from August 22, 2021, to September 29, 2021. A total of 152 papers were imported using agreed-upon search terms, and after careful screening, 17 papers remained.

A considerable share of studies excluded during the title and abstract screening stage were studies that used PROs as data sources but did not assess the barriers and facilitators of using the system. Some of the papers dealt with patient-reported data, but the information was obtained through, for example, a survey on paper or an interview. At that stage, studies related to ePRO implementation rather than barriers and facilitators in using ePRO after the implementation period remained for further full-text review. There was a need to decide when the implementation turned into routine usage, and 3-month implementation period criteria were established.

The main author did the data selection independently. A random selection of 23% (n=25) of full-text papers was reviewed by a different researcher to determine interrater reliability. Interrater reliability was determined using Cohen κ and found to be acceptable at 0.63 (SE 0.25; 95% CI 0.14-1.11). The reviewers had divided opinions on the eligibility of one of the jointly evaluated papers. Therefore, there was a need to clarify when implementation became the routine use of PRO. Multimedia Appendix 1 shows the papers selected according to the criteria of this study. Later in this study, the papers are referenced based on the sequence numbers in the *References* section.

As shown in Table 1, papers addressing the perspectives of HCPs on the barriers to and facilitators of using ePRO data in the treatment of patients with cancer have been published in recent years, while there was no publishing in the early 2010s. The table shows that there is a clear minority of papers describing only ePRO systems in 2020-2021 papers compared to papers focusing on both ePROs and nondigital PROMs.

**Table 1:** The trend of publishing year of included papers.

	2010-2011	2012-2013	2014-2015	2016-2017	2018-2019	2020-2021
All papers	0	0	0	6	1	10
ePRO^a^	0	0	0	5	0	3

^a^ePRO: electronic patient-reported outcome.

### Data Extraction and Analysis

The data on the included papers were extracted as follows: authors, title, year of publication, study objective, cancer type, study design, duration of data collection, study population, administered PROM, ePRO name, sole use of ePRO, barriers, and facilitators. Thematic meta-synthesis for the extracted data was performed by adapting the methods for thematic synthesis described by Thomas and Harden [20]. The verbatim findings of these 17 studies were entered into the Excel (Microsoft Corp) file. Each line of text was coded according to its meaning and content. The authors of the paper independently reviewed the data and grouped them into themes. Themes were then discussed and merged. After several rounds of reviewing the coding, the final 7 descriptive themes for the barriers and facilitators were determined. The 7 themes were summarized into 3 categories that united the themes: work environment, value to users, and suggested features.

## Results

### Overview

Of the total 17 papers, 8 introduced a study in which study participants had used only ePROs [21-28]. In the other 9 papers [29-37], only some of the participants had used ePROs, while others had used nondigital PROMs. The ePRO systems used were AmbuFlex [21,26], OncoQuest [22], Noona [23], the KLIK method [25], and PatientViewpoint [28]. No type of cancer was particularly emphasized, but PROMs were used in different target populations, such as patients with breast, lung, head and neck, prostate, melanoma cancer, and pediatric patients with cancer. The most frequently used PROM was the Edmonton Symptom Assessment Scale (ESAS) [32-34]. Studies were carried out in multiple countries: the Netherlands [22,25,31], Germany [27,29], the United Kingdom [24], the United States [23,28,35,37], Denmark [21,26], Canada [32,34], Australia [33], and 41 other countries [30]. There was no mention of the country where the PROM was implemented in 1 paper [36].

### Thematic Analysis

The following themes were identified from the barriers and facilitators presented in the selected papers: clinical workflow, organization, and infrastructure, value to patients, value to HCP, digital health literacy, data visualization and perceived features, and usability.

#### Clinical Workflow

This theme describes the PROMs’ impact on the smooth running of work tasks in a health care organization, both in the work of individual HCPs and in collaboration with a multiprofessional team.

##### Barriers

The theme was associated with barriers by 8 comments in 13 papers [21-23,25-31,34,35,37]. The lack of integration into EHRs or other hospital systems is highlighted as a barrier in 7 papers [21-23,26,28,29,37]. The impact on the workflow can be a barrier [28] if PROs do not fit into a robust workflow of clinical care [30] and current routines [25]. PROs might delay clinics if it takes too much time for patients to fill them in before physician consultation [27,31,34] or for clinicians to interpret PROMs [31]. The timing of the distribution of the PROM may complicate the clinical workflow [35]. This is especially true if the patient’s consultation with the physician is too far removed from the point of PROM reporting, as it may result in the data being no longer relevant [31].

##### Facilitators

Facilitators of this theme were identified by 8 comments in 7 papers [24,26,29,31,33,35,36]. Easy access to the PROM tool, or data [31,35], specifically via EHR [24,29,33], facilitates the use of PROM. Integration is considered very important [26,36]. The ability of patients to contact the department on time and distribute information automatically to patients is also crucial, which supports the clinical workflow [26]. ePROs are more efficient in data collection, distribution, and preserving data quality than nondigital PROMs [35].

#### Organization and Infrastructure

This theme refers to the existing support systems available in the health care organization, such as the PROM integration into the hospital’s EHR. In addition, the organizational facilitators and barriers to PROM use are discussed under the theme.

##### Barriers

We identified 6 barriers to this theme in 5 papers [21,24,25,30,36]. The ePRO systems are not implemented as routine [21], or ePROs are not integrated as intended [25]. PROMs might lack integration technology with hospital EHR [36]. Some PROs are not systematically collected at different clinics in the hospital [30]. Another barrier is limited access to computers [24]. A complex hospital system could also potentially influence the use of the ePRO system [24].

##### Facilitators

This theme was associated with 4 facilitators in 4 papers [25,29,35,36]. The use of PROM is enhanced if professionals think they are expected to use the ePRO system (normative belief) [25] or if coordinating structures are implemented for PRO processes in hospitals [29] or PROMs improve the coordination of care [36]. In addition, PROMs are more used if the hospital benefits financially from the use, receiving more payments for care [35].

#### Value to Patients

This describes how the use of PROMs affected patient experiences according to HCPs.

##### Barriers

Barriers to value were captured by 14 comments identified in 6 papers [22,28,33,35-37]. Some HCPs expressed concern that patients may not see the point of using ePRO [36,37]; it may be that it takes too much time [22] and that they end up feeling overburdened [33] or experience fatigue by the process [36]. The lack of home access to ePROs [22] and the lack of feedback from physicians [22] were also mentioned. Patients’ inability to complete the PRO can vary based on language [36,37], literacy [35-37], health literacy [33], culture [36], and health status [37]. There can be reporting bias; for example, reporting actual symptoms might not be comfortable for patients [35]. One paper shows that patients might prefer to share their needs directly with HCPs rather than via ePROs [28].

##### Facilitators

There were 6 papers [25,26,33,34,36,37] with 13 comments on the positive views of HCPs on patients’ experiences with PROMs. The perception was that PROM improves patients’ quality of life and satisfaction [37] and empowers them [33]. PROMs make it easier for patients to report their symptoms [34], and they think that they increase treatment adherence and patients’ awareness of their own needs and the resources available for them [36]. HCPs also perceived patients as being better prepared for consultation and more aware of their symptoms. The tool also allows patients to contact the clinic on time. The PROM is especially valuable for patients who are usually unwilling to contact the clinic unscheduled [26]. Professionals deem patients’ opinions important regarding the use of the ePRO [25], and they think it is good that the tool is patient-centered and captures patients’ perspectives [34].

#### Value to HCPs

This theme describes the added value experienced by HCPs in using PROM in their work. In practice, if the comment did not fit other themes identified, it was included in this theme.

##### Barriers

A total of 7 papers [21,23,25,26,28,32,33] included 6 comments on the barriers to this theme. Some users reported that the use of ePROs or PROMs tends to prolong clinic visits [26,32,33]. Some prefer face-to-face communication rather than looking at the computer [28]. Some HCPs already have electronic ways of communicating with their patients [23], and extensive assessments have already been performed [32]. HCPs are skeptical of the value that ePROs add to their interactions [25], as some clinicians rate the importance of symptoms differently than patients [21].

##### Facilitators

The theme included 27 facilitating comments in 13 papers [21,22,24-28,31-36]. The potential time saved was highlighted in 3 papers [27,31,36], and 1 paper commented on how the consultation was shorter with ePRO use [21]. The ability to improve communication between patients and clinicians is very important [21,36]; the information seems to be more discussable [25], as it allows for the comparison of symptoms and treatment evaluation [33]. PROMs are perceived as enhancing consultation efficiency [34] and are considered a systematic and measurable method for assessing patient needs [32]. They are sometimes helpful and sometimes confirmatory [28] and have value [32].

PROM appears to stimulate multidisciplinary teamwork [31]. There is an opportunity with ePRO to develop follow-up referrals to better meet the needs of individual patients [22]. ePRO is a useful addition to the clinical management of patients [24] and should be used as a basis for patient-clinician consultation and as an added benefit for the consultation [26]. ePRO helps clinicians understand patients’ experiences of recovery and monitoring symptoms [24] and prioritize patients’ problems [26]. There is no similar need for explanatory information when looking at patient results [28]. PROM enhances clinicians’ awareness of patients’ needs [36], patient-centered care [35], and knowledge of patients’ health-related quality of life [22,25]. Diagnosis-based ePRO instruments are facilitators for clinicians [35].

#### Digital Health Literacy

This theme refers to the competence, opportunity to develop, and ability of HCPs to work in a digital workplace.

##### Barriers

There were 6 papers [21,25,27,28,30,31] with 13 comments associated with barriers to this theme. Barriers ranged from understanding the basics of PROM systems [31] (how to log on, the aim of the system, how the data are presented, and how the ePRO is used in communication with patients [21]) to more systemic issues (lack of support from colleagues [25], management [30], and local PRO experts [30]). The absence of technical support [21,31] and high administrative burden [31] are often present. There is also a lack of knowledge on some assessed data in ePRO [27] and a need to have better indications of what certain scores mean [28]. Uncertainty about how to choose an appropriate PROM is also a barrier [30].

##### Facilitators

The theme includes 3 facilitating comments in 2 papers [25,33]. Providing sufficient education on the use of ePRO systems [25] and identifying patients’ symptoms through PROMs [33] are valuable. For the development of hospital service delivery, PROMs provide information by highlighting the symptom groups of the patient population [33].

#### Data Visualization and Perceived Features

This theme describes needs regarding the representation of data and information with visual elements, such as charts and graphs, intended to make it easier for the user to understand the data, such as trends and outliers.

##### Barriers

The theme was associated with 8 barriers mentioned in 6 papers [27,28,30,34-36]. The publications described certain features that are lacking in most cases, such as cost-effectiveness data [30] and automatic referrals to follow-up treatment [36]. More answering options are desired to make the questions appropriate for all patient situations [27]. Some users hope for other symptom options [34], and some prefer features that could flag high symptom scores [28]. More functionalities [28] and well-designed features are needed to avoid information overload [35]. Graphs are preferred over tables [28].

##### Facilitators

Facilitators of the theme were identified by 9 comments in 8 papers [21,23,25,27-29,31,35]. Good data visualization was mentioned in 2 papers [27,35], and 5 papers placed special emphasis on graphical representation [21,23,25,29,35]. The facilitators include clear reports that are easy to comprehend [29,31] and are done using color schemes and cutoff points [29]. One figure should show all the measured data [29]. Predesigned templates with easy-to-remember phrases [35] and email reminders sent to patients from the system [28] facilitate the use of ePROs.

#### Usability

This refers to the aspect that affects how easy it is to use a PROM.

##### Barriers

Two papers [29,34] mentioned barriers to the usability of the PROMs theme. The barriers include a lack of coordinating structures of the PROM between wards [29] and a lack of design specific to the cancer populations [34].

##### Facilitators

The theme had 6 facilitators identified in 6 papers [21,23,25,31,34,35]. Three studies that presented only ePRO use [21,23,25] raised the issue of being easy to use in 7 comments. Systems are easy to use [21,23] or are not too complicated to use [25]. Two papers that included both ePRO and nondigital PRO use presented the experience of the ease of use of PROM [31,35] and the other one also as an actionable tool [35]. One paper agreed that the PRO tool is a good way to start assessing patients’ symptoms [34]. Better customizability of the questionnaire improves usability [35] and displays results in such a way that they are easy to understand [31].

### Takeaway Points

#### Work Environment

- The use of ePRO should be adapted to the workflow of the clinic to ensure the smooth operation of the system.
- The ePRO should be completed by the patient at the time most valuable to the timing of the patient’s treatment.
- The use of ePRO should be integrated into all hospital settings so that it works and is in use in all hospital units.
- The ePRO should be interoperable with the hospital’s EHR.

#### Value to Users

- ePRO is valuable to HCPs in symptom management, but it is important to strive to reduce the potential bias between patients’ and physicians’ symptom assessments.
- It is important to consider the patient’s ePRO notes and give feedback to the patient at the clinical visit, and to have a system to capture patients’ perspectives.
- ePRO facilitates patient-clinician communication, but it must not limit patient-clinician face-to-face communication. It is valuable for the clinician to check the patient’s ePRO entries before the patient visits.
- Providing a means for patients to access the ePRO is very important. Options should be available that take into consideration language, literacy, health literacy, culture, and health status.
- The content of PROMs is more valuable to users if it is designed specifically for different cancer indications.
- Users should understand how to use the system well enough and understand its purpose.
- The electronic format of ePROs enables statistical analysis and visual representation of data, which can lead to decision support and improved patient outcomes.
- HCPs should get technical support and support from colleagues, management, and local PRO experts to use the system.

#### Suggested Features

- Summaries and overviews displaying measured data can enhance the understanding of PROMs.
- Special attention should be paid to the visualization of data, favoring graphic presentation.
- There should be a feature to flag high symptom scores to make them more noticeable to HCP.
- Color schemes and cutoff points make the user interface easier to comprehend.
- Predesigned templates help select the platform most appropriate to patients’ treatment.
- The system should show cost-effectiveness data of the treatment to the HCP.
- The system should automatically create referrals for follow-up treatment.
- Patients should receive reminders to use the system.
- Patients’ applications should have more response options for patients in different situations, such as with additional symptoms.

## Discussion

### Principal Results

This mapping study identified multiple barriers and facilitators to using ePROs for cancer care. The highlights of these are presented in condensed form as takeaway points for easy reading in categories such as work environment, value to users, and suggested features. Our work exposes the need for future studies on the use of ePROs compared to studies on the use of paper PROs.

The findings of this study strongly support the active integration of ePRO into the surrounding work environment. Earlier knowledge emphasizes the importance of functional workflow [12]. This leads to the notion that ePRO’s operations should be integrated into the hospital workflow to allow users to experience ePROs’ seamless use in the hospital setting. Based on this, it could be valuable to optimize the use of ePRO together with hospital operations at the time of the implementation of the ePRO. This study also revealed the need for ePRO integration in different hospital units. If the same ePRO is in use in different specialties, the system data could be better used in the multidisciplinary care of patients. Further, ePRO integration into the EHR in the hospital was highlighted in 9 of the 17 papers analyzed, which is well-aligned with prior works [9,12,14].

There are different lines of thought regarding the moment in which ePROs are best deployed for patients. The use of web-based ePRO at home may be advantageous, as the memory of the symptoms may be fresher, whereas use during the clinical visit may increase overall use. If patients complete the ePRO at the beginning of the clinical visit, it may help reduce the fear of losing personal contact with professionals and lower digital literacy needs [38]. Allowing patients to choose when to complete the ePRO could be a good compromise. Finally, when the patient is at the doctor’s office, patient-clinician face-to-face communication is needed instead of clinicians looking at the computer screen to look at the ePRO information [11]. This is also supported by Gilligan et al [39] in their consensus guideline of patient-clinician communication, where they recommend considering using PROs to prepare the patient visit.

This study emphasizes multiple benefits for patients and the importance of patients’ opinions for HCPs regarding the use of PRO systems [25]. These findings are consistent with Roberts et al [14]. Their study demonstrates the desire of HCP to be active in implementing PROMs into routine oncology care if patients benefit from the use and if the use of PROM improves health care. According to these studies, it is important to inform HCPs about patients’ views and what facilitates patients’ use of ePRO. It may strengthen HCPs’ experience of the relevance of ePROs and improve the user experience if patients have positive experiences and feel that they will benefit from using ePRO.

This study highlighted the potential of ePRO systems to help develop the treatment of patients by using the data generated by the system, for example, about the symptom groups of the patient population [33]. A recently published study has also shown the important role of ePRO data in examining the benefits and efficacy of new innovative treatments [40]. Based on this, one can assume that the benefits of ePRO are wider than monitoring the treatment of an individual patient. ePRO data also play an important role in the development of treatments for patients with cancer.

The number of comments on the different themes shows how strongly the ePRO value for patients and HCPs facilitates the use of ePROs. Of 128 comments that presented barriers or facilitators, 60 described the value to patients or HCPs. In other words, for “value to users,” 25 of the comments concerned “usability” and “data visualization and perceived features” of the systems. Although this proportion is not as prominent compared to the “value to users” theme, there are some explicit features mentioned that should be considered while developing the system to become more practical for the users.

According to this study (Table 1), only a few studies are available on the barriers and facilitators of ePRO use, including both ePRO and nondigital PROMs. In total, 8 papers described studies that used ePROs alone; the other 9 papers had both electronic and nondigital PRO data. Interestingly, studies on ePRO only have declined in recent years. The smaller number of ePRO studies could suggest that using ePRO has not supplanted the use of nondigital PROs. Thus, there is still a need for the knowledge and development of ePROs.

### Limitations

This study has several limitations. As systematic mapping studies rely on the selection process to identify relevant studies, there is a risk of bias that can affect the results and conclusions of the study. Search results are only as current as the date of the last search performed. The quality of the studies included varies greatly, and the limitations of individual studies can affect the overall results and conclusions of the mapping study.

Further, this kind of study does not allow for in-depth analysis of individual studies or a detailed synthesis of the findings. However, systematic mapping studies are helpful to provide an overview of the existing literature, which was the goal of this study.

We decided to focus on the barriers and facilitators experienced by professionals. Patients’ experiences were excluded, except when reported by professionals. This resulted in a one-sided perspective on the use of ePRO systems. Other stakeholders’ opinions were not discussed. We grouped the barriers and facilitators compiled from the analyzed papers into themes and themes into categories. These themes and categories are the researchers’ views on the issue. Some papers included comments from respondents who had never used ePRO systems. We could not ascertain that those responses were different from responses by users who had experience using ePROs.

In the data screening phase, we made efforts to remove the barriers and facilitators identified in the paper version of PROs. Thus, it is possible that the comments from the paper version of PROMs and ePROs were partially mixed.

Studies that demonstrated the barriers and facilitators of the implementation phase of PROM were excluded from the study. The decision to limit the implementation phase to 3 months may have indirectly affected our findings. Although this decision helped in classifying and screening the results, relevant papers might have been excluded.

### Conclusions

In this study, we provided a broad overview of the barriers and facilitators affecting the use of ePROs. Our work focused on how the working culture and service integration affects the success of ePRO. A greater understanding of barriers and facilitators is useful to software developers and clinical research organizations to create smoother implementations. We found that there are multiple ways to develop ePROs and their working environments to meet the needs of HCPs. They can be summarized into 3 categories: work environment, value to users, and suggested features. The takeaway points detail the findings of this study.

### Future Research

Based on this study, there is still a lack of information on the national and international knowledge of ePROs. Since there are only a few studies on fully electronically completed PRO data, future research should explore the barriers and facilitators of using ePROs, specifically in organizations where users have sufficient experience using ePROs. As this study is limited by the literature currently available in the selected databases, further work may expand on the knowledge by including additional sources and terms. Future work could also focus on exploring how the implementation of ePROs may affect the patient’s journey through the health care system. It would also be interesting to understand whether more usability and features are required of an ePRO than of a paper PROM, given that it is possible to implement features beyond those of paper versions.

It would also be interesting to explore in more detail how common it is that patients rate their symptoms differently than physicians treating them and how patients’ personal experiences are considered in treatment. Can ePRO be further developed to identify rating differences? Would it help if ePRO were to add more detailed parameters to the symptoms? Concerning challenging symptom descriptions, research may be conducted to determine which symptoms or symptom descriptions differ most in terms of patient and physician perceptions and, based on this, develop an ePRO to highlight a potential bias.

## Appendix

Multimedia Appendix 1 Searches in databases.

## Abbreviations

EHR: electronic health record
ePRO: electronic patient-reported outcome
ESAS: Edmonton Symptom Assessment Scale
HCP: health care professional
PRO: patient-reported outcome
PROM: patient-reported outcome measure
