# Supplementary material for: Perceived Barriers and Facilitators in Using Patient-Reported Outcome Systems for Cancer Care: Systematic Mapping Study
Source: JMIR Cancer. 2023 Jun 28;9:e40875. doi: 10.2196/40875 (PMC10365581; doi:10.2196/40875)
Supplement: Multimedia Appendix 1 [file cancer_v9i1e40875_app1.docx]

# Multimedia Appendix 1. Searches in databases.
